# Bilobed Flap for Reconstruction of a Large Keratoacanthoma of the Thumb

**DOI:** 10.1080/23320885.2019.1634477

**Published:** 2019-07-05

**Authors:** Özgür Agdoğan, Rahşan Karaçal

**Affiliations:** a Department of Plastic Reconstructive and Aesthetic Surgery, Tekirdag Namik Kemal University Faculty of Medicine, Tekirdag, Turkey;; b Tonya Vocatıonal School Health Care Servıces, Karadeniz Technical University, Trabzon, Turkey

**Keywords:** Thumb, large keratoacanthoma, bilobed flap

## Abstract

We present the bilobed flap principle which can be used to reconstruct of large defects located on the dorsum of the thumb. The flap provides similar texture, colour and thickness as adjacent hand skin. The final results were excellent both functionally and cosmetically.

The dorsum of the hand is one of the most frequent sites for skin lesions. Primary closure of the defects, following a convenient excision of such lesions is not feasible for most of the cases [1]. According to the reconstructive ladder, skin grafting and local flaps are the next steps after the primary closure techniques [2].

The bilobed flap was first described by Esser in 1918 for use in nasal tip defect reconstruction. In 1953, Zimany reported his experience with bilobed flaps in several anatomical sites including the face, trunk and sole of the foot. Many authors have also mentioned the importance of this flap over time and confirmed its usefulness. That is, the bilobe flap has a history of about 100 years [3].

Local flaps, which are based on ‘like with like’ principle, are the best options for reconstruction of the defects of the hand [1]. Here, we present reconstruction of a dorsal thumb defect occurred as a result of a large keratoacanthoma excision with a bilobed flap.

A 66-year-old left-handed woman presented us suffering from a lesion located on her left hands thumb dorsa which increase its size slowly in 2 months. The lesion was causing pain and loss of function of the thumb. On physical examination, there was a 2 cm × 1.5 cm × 1 cm sized dome-shaped lesion located on distal half of the first metacarpal and metacarpophalangeal joint (MCPJ) (Figure 1).

**Figure 1.**
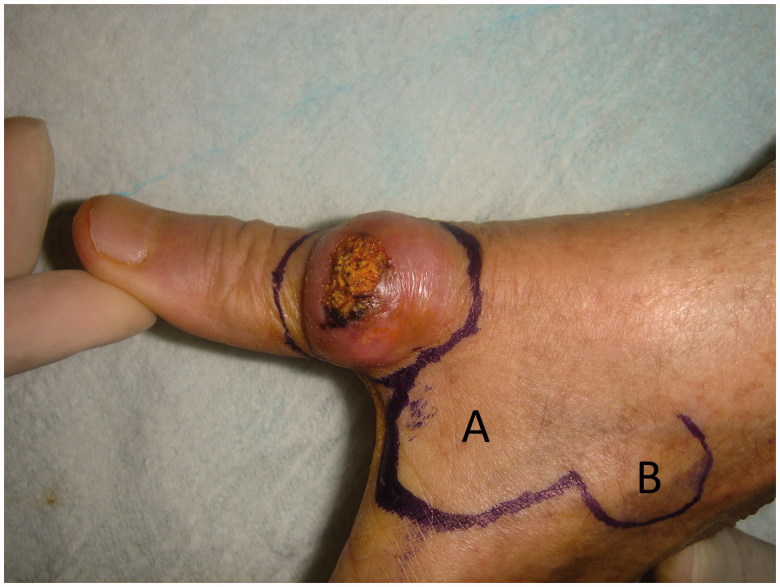
Preoperative view of the lesion and planning of bilobed flap.

The management of the case was challenging due location and size of the lesion. We aimed to achieve both aesthetic and functional reconstruction, and decided to use a bilobed flap to closure the 3 cm × 2 cm sized defect.

The surgical excision of the lesion was done with local anaesthesia which is infiltration of the area with %2 lidocain with adrenaline. Excision of the lesion was planned, then the first flap (A flap), which was slightly narrower than the defect, was designed from the dorsal skin of the 2nd metacarpal area. The second flap (B flap) was half the width of the first flap and angles between the lobes were 90°. The second flap designed with an elliptical tip to facilitate closure of its resulting defect (Figure 1). Following the excision of the lesion, the flaps were raised using a scissors-spreading technique and wide undermining was used to reduce tension and pincushion effect. The flaps then were transposed into the defects and sutured in place (Figure 2).

**Figure 2.**
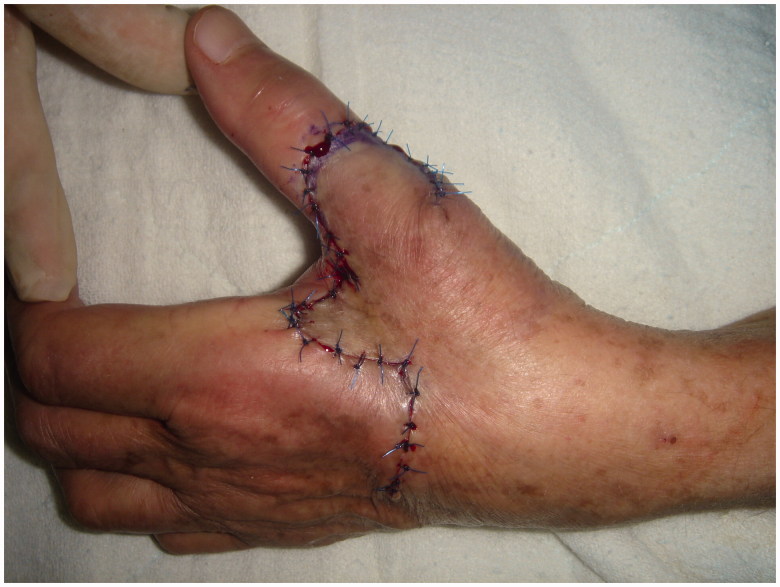
Postoperative view of reconstruction.

The histopatology of the lesion revealed that it was keratoacanthoma with negative surgical margins. No complications were observed postoperatively. The patient rehabilitated for 4 weeks just after the removal of the stitches at the 2nd week. The reconstructions final outcome was cosmetically excellent and resulted without functional compromise after 6 weeks post-operatively.

Maintaining durability and sensibility of the hand, while protecting it from side effects of the surgery such as contractures and joint stiffness, are the primary objectives of hand reconstruction procedures [2]. The bilobed flap is a double transposition flap commonly used in reconstruction of areas where skin is less mobile such as nasal dorsum reconstruction. On the other hand, the usage of bilobed flap for reconstruction of dorsum of the thumb is not that common. Alternatively, the defect might be reconstructed by full thickness skin graft, but this method would cause a donor site scar and possible MCPJ contracture.

Bilobe flaps are generally used for nose and face reconstruction. The blood supply of the facial region is quite high and there is enough skin tissue for reconstruction. However, there is not much skin tissue in the hands and especially the fingers. There is no routine bilobe flap between the flap options in the fingers. We thought that it would be beneficial to use the bilobe flap option in the hands and especially the fingers.

In conclusion, here we present the bilobed flap principle which can be used to reconstruct of large defects located on the dorsum of the thumb. The flap provides similar texture, colour and thickness as adjacent hand skin. The final results were excellent both functionally and cosmetically.
